# Ruxolitinib alleviates DSS-induced acute ulcerative colitis by inhibiting STAT1 phosphorylation and reducing MDSC infiltration

**DOI:** 10.3389/fphar.2025.1572534

**Published:** 2025-08-29

**Authors:** Lu Xu, Fangyue Xu, Qinghua Yao, Vincent Kam Wai Wong

**Affiliations:** ^1^ Dr. Neher’s Biophysics Laboratory for Innovative Drug Discovery, State Key Laboratory of Quality Research in Chinese Medicine, Faculty of Chinese Medicine, Macau University of Science and Technology, Macau, China; ^2^ The Second Clinical Medical College, Zhejiang Chinese Medical University, Hangzhou, China; ^3^ The Second Affiliated Hospital of Zhejiang Chinese Medical University, Xinhua Hospital of Zhejiang Province, Hangzhou, Zhejiang, China

**Keywords:** ruxolitinib, DSS, acute ulcerative colitis, myeloid-derived suppressor cells (MDSCs), STAT1 phosphorylation

## Abstract

**Objective:**

This study aimed to evaluate the therapeutic effects of Ruxolitinib, a JAK1/2 inhibitor, on DSS-induced acute colitis in mice, with a focus on its impact on disease activity, inflammatory responses, modulation of myeloid-derived suppressor cells (MDSCs), and regulation of the JAK/STAT1 signaling pathway.

**Methods:**

Acute UC was induced in C57BL/6 mice by administering a 2.5% DSS solution. Mice were randomly assigned to three groups: the blank group (no DSS), the model group (DSS only), and the Ruxolitinib-treated group (DSS +30 mg/kg Ruxolitinib by gavage for 14 consecutive days). Body weight, disease activity index (DAI) scores, spleen weight, and colon length were measured. Spleen index and the spleen weight-to-colon length ratio were calculated. Flow cytometry was used to assess the proportion of MDSCs in the blood. *In vitro*, CCD841 and Jurkat cells were pretreated with 50 IU/mL IFN-γ for 2 h, followed by 24-h treatment with Ruxolitinib. PCR array analysis was performed to identify transcriptional changes in JAK-STAT pathway-related genes. Electrophoretic mobility shift assay (EMSA) and Western blot were used to investigate the inhibition of STAT1 activation and phosphorylation.

**Results:**

*In vivo*, DSS-induced acute colitis in the model group, and Ruxolitinib treatment significantly alleviated colitis as evidenced by reduced body weight loss (p < 0.05), decreased DAI scores in the later stages (p < 0.05), a lower spleen index (p < 0.05), increased colon length (p < 0.01), and a reduced spleen weight-to-colon length ratio (p < 0.05). Flow cytometry revealed a significant reduction in the proportion of CD11b^+^ Gr-1^+^ MDSCs in the blood of the Ruxolitinib group compared to the model group (p < 0.01). *In vitro*, PCR array analysis showed that Ruxolitinib notably downregulated the transcription of several JAK-STAT pathway-related genes, including B2M, IRF1, RQ1, SOCS1, STAT1, and STAT3, with STAT1 showing the most pronounced changes. EMSA and Western blot analysis confirmed that Ruxolitinib effectively inhibited IFN-γ-induced STAT1 activation and phosphorylation in a dose-dependent manner.

**Conclusion:**

Ruxolitinib effectively ameliorated DSS-induced acute colitis by reducing inflammation, modulating MDSC levels, and inhibiting STAT1 activation. These findings suggest that Ruxolitinib could be a promising therapeutic agent for UC, targeting both the immune response and the JAK/STAT1 signaling pathway.

## 1 Introduction

Ulcerative colitis (UC) is a chronic, relapsing inflammatory bowel disease (IBD) characterized by inflammatory damage to the colonic mucosa (Lamb et al., 2019). The pathogenesis of UC is complex and multifactorial, involving interactions between genetic susceptibility, immune dysregulation, and environmental factors. Immune-mediated disruption of intestinal homeostasis is recognized as a central mechanism driving the inflammatory response in UC (Ramos and Papadakis, 2019). In this process, myeloid cells proliferate abnormally and differentiate into myeloid-derived suppressor cells (MDSCs), which exhibit immunosuppressive and anti-inflammatory properties. These cells play a pivotal role in immune imbalance and inflammation-associated carcinogenesis in UC (Ibrahim et al., 2018). MDSCs contribute to the pathogenesis of UC by interfering with colonic mucosal repair, maintaining an inflammatory microenvironment, secreting immunosuppressive molecules, inhibiting T cell function, and promoting inflammation spread (Guan et al., 2013).

The Janus kinase (JAK)-signal transducer and activator of transcription (STAT) signaling pathway is crucial in regulating immune cell activation and mediating pro-inflammatory cytokine responses (Chaimowitz et al., 2024). Ruxolitinib, a JAK1/2 inhibitor, has demonstrated therapeutic potential in various autoimmune diseases by blocking the JAK-STAT pathway (Papp et al., 2023; Zhang et al., 2022). However, its role in UC remains underexplored. Previous studies suggest that the JAK-STAT pathway plays a critical role in regulating T cell differentiation and MDSC function during UC-related inflammation (cordes et al., 2020). Therefore, investigating the immunoregulatory effects of Ruxolitinib in acute UC, particularly its impact on MDSC populations and the JAK/STAT signaling pathway, holds significant promise.

This study aims to evaluate the regulatory effects of Ruxolitinib on MDSC proportions in the blood of mice with DSS-induced acute UC, and to explore its influence on the phosphorylation of STAT1 induced by IFN-γ in various cell types. The findings from this study may offer new insights into the potential of Ruxolitinib as an immunotherapeutic agent for UC.

## 2 Materials and methods

### 2.1 Reagents

DSS was purchased from MP Biomedicals; Ruxolitinib was purchased from Selleck; The following antibodies were used: APC-conjugated anti-CD11b and PE-conjugated anti-Gr-1 (BioLegend), STAT1 and phosphorylated STAT1 (Tyr701) (Proteintech), phosphorylated STAT1 (Ser727) (Cell Signaling Technology). Stat1 Gel Shift Oligonucleotides were purchased from Santa Cruz Biotechnology. Other reagents included rabbit secondary antibody and red blood cell lysis buffer (Biotool), 3,3′,5,5′-Tetramethylbenzidine dihydrochloride hydrate (Shanghai Shenggong Biotech), BCA Protein Assay Kit (Solarbio), Trizol, SuperScript III Reverse Transcriptase, and SuperArray PCR Master Mix Kit (ThermoFisher), and RT2 Profiler™ PCR Array Human JAK/STAT Signaling Pathway (Qiagen).

### 2.2 Equipment

The following equipment was used: refrigerated high-speed centrifuge (Eppendorf), vortex mixer (Scientific Industries), flow cytometer (BD), ABI 7500 Real-Time PCR System (ThermoFisher), and ChemiDoc imaging system (Bio-Rad).

### 2.3 Animal model and preparation

Male C57BL/6 mice (5–6 weeks old) were purchased from Shanghai SLAC Laboratory Animal Co., Ltd. Mice were housed in an SPF-level animal room under controlled conditions with a 12-h light/dark cycle. After 1 week of adaptive feeding, the mice were randomly divided into three groups: blank group, model group, and Ruxolitinib group (n = 6 per group). Acute UC was induced by administering 2.5% DSS solution for 10 days, followed by 5 days of purified water. TheRuxolitinib group received 30 mg/kg Ruxolitinib (solvent composition: 10% DMSO, 40% PEG300, 5% Tween-80, 45% physiological saline) daily via gavage for 14 days, while the model group received an equivalent volume of vehicle. On day 15, mice were euthanized, and blood, spleen, and colonic tissues were collected for analysis.

### 2.4 Evaluation of DSS-Induced colitis severity

#### 2.4.1 Assessment of disease activity through daily DAI scoring

Mice were monitored every 2 days for body weight, food intake, mental status, and fecal characteristics. Disease activity was assessed using the DAI score, as previously described (Guo et al., 2022), showed in Table 1.

**TABLE 1:** Disease activity index scoring standard.

Score	Body weight loss (%)	Fecal consistency	Hemorrhage/Occult blood
0	0	Normal feces	-
1	1-5	-	+
2	6-10	Semi-formed stool	++
3	11-15	-	+++
4	>15	Diarrhea	Visible blood in stool

#### 2.4.2 Evaluation of systemic inflammation via spleen index and colon length measurement

On day 15, mice were euthanized, and blood samples were collected by enucleating the eyeballs and stored in 1.5 mL anticoagulant tubes for flow cytometry analysis. The spleen was excised, weighed, and the spleen index was calculated as the ratio of spleen weight to body weight. The entire colon (from cecum to anus) was excised, and its length was measured. The spleen weight-to-colon length ratio was also calculated.

#### 2.4.3 Quantification of MDSC levels in mouse blood using flow cytometry

A total of 100 μL of blood was drawn from each mouse, and 2 mL of red blood cell lysis buffer was added. The mixture was gently shaken for 10 min and centrifuged at 400 *g* for 4 min at 4 °C. The supernatant was discarded, and the cell pellet was resuspended in 100 μL of flow cytometry staining buffer (PBS +3% FBS). A blank control tube was prepared, and an antibody cocktail (1:100 dilution) was added to each tube. Tubes were incubated on ice in the dark for 30 min, followed by centrifugation at 400 *g* for 2 min at 4 °C, and two washes with staining buffer. The cells were resuspended and analyzed by flow cytometry.

### 2.5 Transcriptomic profiling of JAK/STAT pathway via PCR array

The CCD841 and Jurkat cells (cultured in RPMI 1640 + 10% FBS, 100 U/mL penicillin/streptomycin at 37 °C/5% CO_2_) were either unstimulated or stimulated with IFN-γ, and total RNA was extracted using Trizol reagent. Total RNA was mixed with oligo (dT) and dNTPs, and incubated at 65 °C for 5 min. Reverse transcription was carried out by adding reverse transcription mix, followed by incubation at 50 °C, and enzyme inactivation at 70 °C to prepare cDNA.

The cDNA was diluted and mixed with SuperArray PCR Master Mix, and 20 μL of the mixture was added to each well of the RT^2^ Profiler™ PCR Array Human JAK/STAT Signaling Pathway. Real-time PCR was performed as follows:

1. Polymerase activation/initial denaturation: 95 °C for 10 min.
2. Amplification cycles: 40 cycles (95 °C for 15 s, 60 °C for 1 min, collecting fluorescence signal);
3. Melting curve analysis: as per instrument requirements.

### 2.6 Electrophoretic mobility shift assay (EMSA) of p-STAT1 activation

For EMSA, CD841 cells (cultured in DMEM +10% FBS, 100 U/mL penicillin/streptomycin at 37 °C/5% CO_2_) were either unstimulated or stimulated with IFN-γ. Nuclear extracts were prepared as previously described (Sanguino et al., 2004), with the addition of protease inhibitors leupeptin (10 μg/mL), antipain (5 μg/mL), pepstatin (5 μg/mL), and phenylmethylsulfonyl fluoride (1 mM). A total of 6 μg of nuclear extract was incubated with a STAT1 probe (5′-CAT​GTT​ATG​CAT​ATT​CCT​GTA​AGT​G-3′), along with IgG, Ruxolitinib, or specific antibodies. The reaction products were separated by electrophoresis on a 5% nondenaturing polyacrylamide gel in TBE buffer.

### 2.7 Western blot

Cells in the logarithmic growth phase were seeded at 5 × 10^5^ cells/well in a 6-well plate and incubated overnight. The cells were then stimulated with 50 IU/mL IFN-γ and harvested at different time points (0 min, 2 min, 10 min, 1 h, 2 h, 8 h, 1 day, 2 days, and 3 days). Total protein was extracted, and protein concentration was determined using the BCA method. Protein samples were denatured at 95 °C for 10 min and loaded (20 μg of protein per well) onto a 10% SDS-PAGE gel. Electrophoresis was performed at 80–100 V for 1 h. Proteins were transferred to PVDF membranes at 200 mA for 1.5 h at 4 °C. Membranes were blocked with 5% non-fat milk for 1 h, followed by overnight incubation with primary antibodies at 4 °C. Afterward, secondary antibodies were applied, and protein bands were visualized using the ChemiDoc imaging system.

In Ruxolitinib intervention experiments, the same cell seeding method and IFN-γ concentration were used. After 8 h of IFN-γ pretreatment, Ruxolitinib was added at varying concentrations for 24 h. Cells were then collected, and proteins were extracted for Western blot analysis as described above.

### 2.8 Statistical analysis

Data were analyzed using GraphPad Prism 9.0 software. Differences between groups were assessed using independent samples t-tests, with significance set at *p* < 0.05. All results are expressed as the mean ± standard deviation (SD).

## 3 Results

### 3.1 Effect of ruxolitinib on activity and DAI index in DSS-Induced acute colitis mice

**FIGURE 1:**
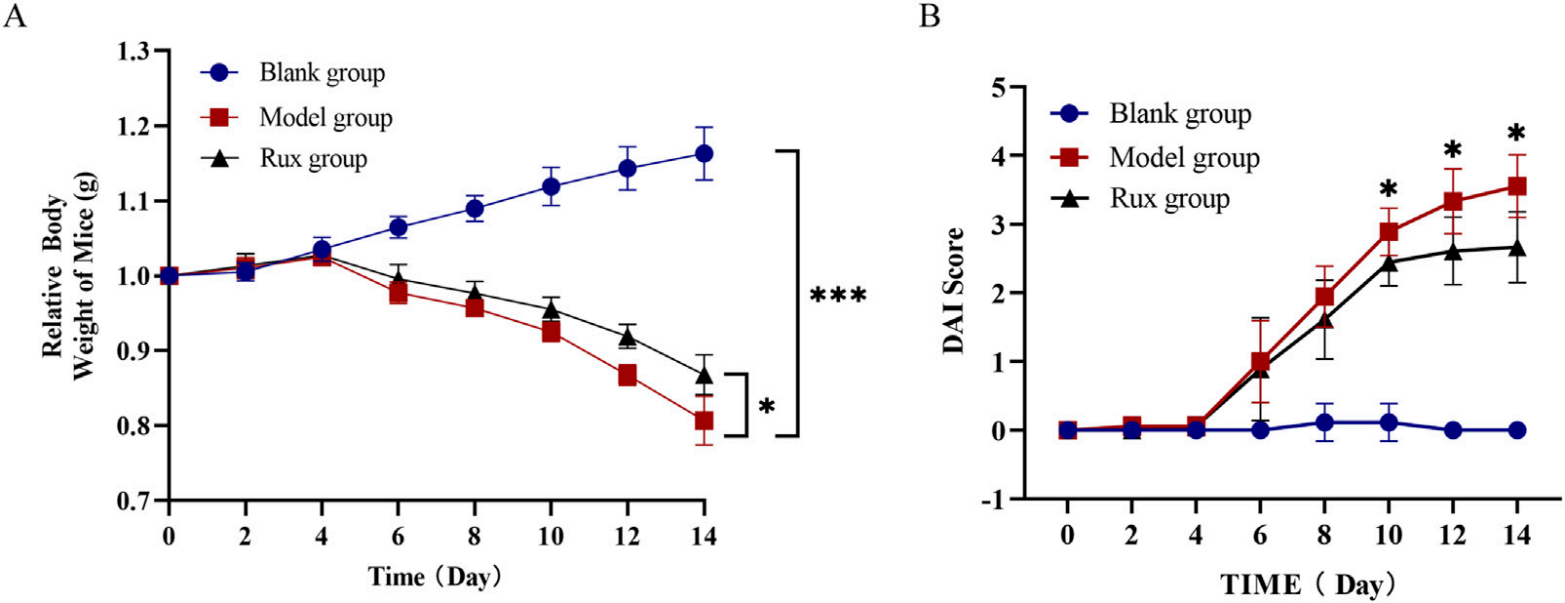
Effect of ruxolitinib on body weight and disease activity index (DAI) in dextran sulfate-induced acute ulcerative colitis (UC) mice. **(A)** Effect of ruxolitinib on body weight changes in DSS-induced UC mice. **(B)** Effect of ruxolitinib on the DAI score in DSS-induced colitis mice. *P < 0.05. ***P < 0.001. Data = mean ± SEM, n = 6/group. The blank group received no modeling or treatment. RUX: ruxolitinib.

The DSS-induced colitis model displayed typical signs of acute inflammation and ulceration. By day 4 after DSS administration, mice began showing symptoms such as lethargy, reduced appetite, and decreased water intake. By day 6, significant weight loss (Figure 1A) and a marked increase in the disease activity index (DAI) score (Figure 1B) were observed, with some mice developing blood in their stool compared to the blank group.

Ruxolitinib treatment significantly alleviated these symptoms. Mice in the Ruxolitinib group exhibited reduced body weight loss compared to the model group, with a statistically significant difference observed starting from day 10 (Figure 1A, p < 0.05). Additionally, the DAI index in the Ruxolitinib-treated group remained lower than in the model group starting from day 8, although the difference was not significant at that time. Notably, after day 10, the DAI index in the Ruxolitinib group showed a significant reduction, indicating the therapeutic effect of Ruxolitinib in the later stages of treatment (Figure 1B, p < 0.05).

### 3.2 Effect of ruxolitinib on spleen weight to colon length ratio in mice

**FIGURE 2:**
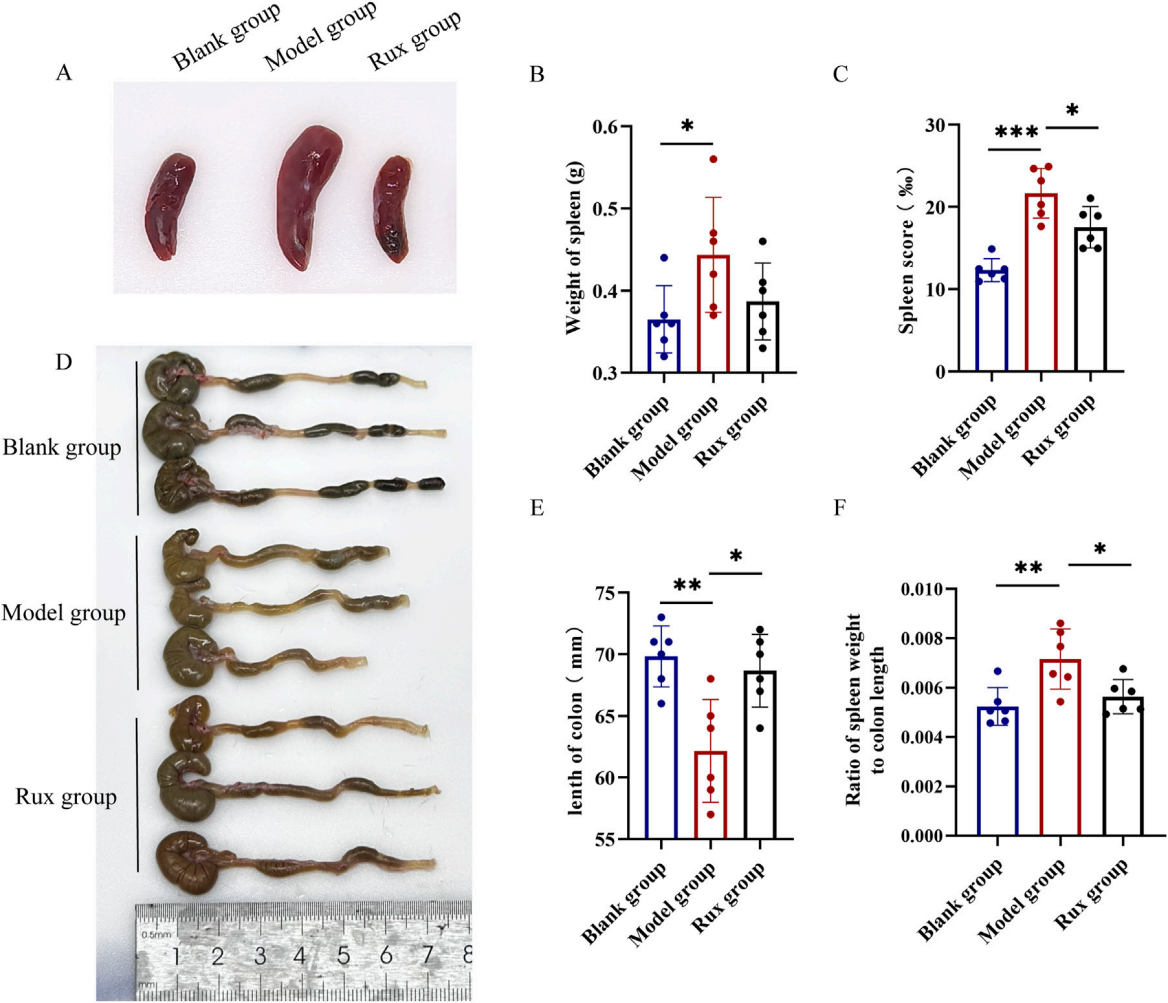
Effect of ruxolitinib on spleen weight to colon length ratio in mice. **(A)** Representative image of splenic tissue. **(B)** Bar graph showing spleen weight. **(C)** Bar graph showing spleen index. **(D)** Representative morphological image of colonic tissue. **(E)** Bar graph showing colon length. **(F)** Bar graph showing the ratio of spleen weight to colon length. *P < 0.05; **P < 0.01. ***P < 0.001. Data = mean ± SEM, n = 6/group. RUX: ruxolitinib. The blank group received no modeling or treatment.

Spleen enlargement is a common indicator of inflammation or infection, while colon length serves as a clinical marker of the severity of acute ulcerative colitis. To evaluate the therapeutic effect of Ruxolitinib, we measured the spleen weight to colon length ratio. Mice in the model group exhibited significant spleen enlargement and congestion compared to the blank group, while these symptoms were notably alleviated in the Ruxolitinib-treated group (Figure 2A). The average spleen weight and spleen index in the model group were significantly higher than those in the blank group. Although the average spleen weight in the Ruxolitinib-treated group was lower compared to the model group (Figure 2B), this difference did not reach statistical significance. However, the spleen index was significantly reduced in the Ruxolitinib group (*p* < 0.05; Figure 2C).

The model group exhibited pronounced colon edema, thickening, bleeding, and disorganized feces, as observed through visual inspection of the colon, compared to the blank group. In contrast, the Rux group displayed significant alleviation of these changes (Figure 2D). The average colon length in the blank group was 69.8 ± 2.3 mm, while in the model group, it was 62.2 ± 4.2 mm. In the Ruxolitinib group, the average colon length was 68.7 ± 2.9 mm, significantly longer than that observed in the model group (*p* < 0.01; Figure 2E). Furthermore, DSS cause a significant increase in the spleen weight to colon length ratio, while Ruxolitinib treatment significantly reduced this ratio (*p* < 0.05; Figure 2F).

### 3.3 Inhibitory effect of ruxolitinib on MDSC levels in mouse blood

**FIGURE 3:**
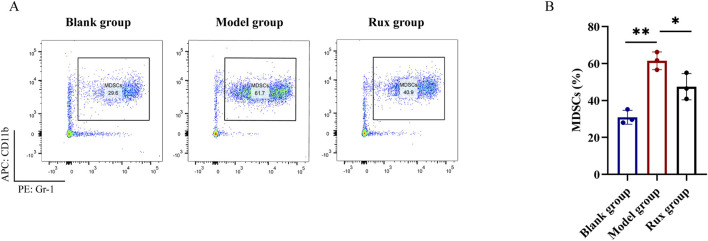
Changes in myeloid-derived suppressor cells (MDSCs) cell population in blood after ruxolitinib intervention. **(A)** Representative flow cytometry analysis of the MDSC population. **(B)** Bar graph analysis of MDSC proportions. RUX: ruxolitinib. *P < 0.05. **P < 0.01. The blank group received no modeling or treatment.

Flow cytometry was performed to detect the proportion of MDSCs (CD11b^+^ Gr-1^+^) in the blood of mice. The blank group exhibited an MDSC level of 30.9% ± 3.8%, while the model group showed a significant increase in MDSC levels, reaching 61.5% ± 4.8%. In contrast, the Rux group demonstrated a reduction in MDSC levels to 47.5% ± 7.1% (Figure 3A). Statistical analysis revealed that the proportion of MDSCs was significantly higher in the model group compared to the blank group (p < 0.01), and Ruxolitinib treatment significantly lowered MDSC levels compared to the model group (p < 0.01; Figure 3B).

### 3.4 PCR array results for the JAK/STAT signaling pathway

**FIGURE 4:**
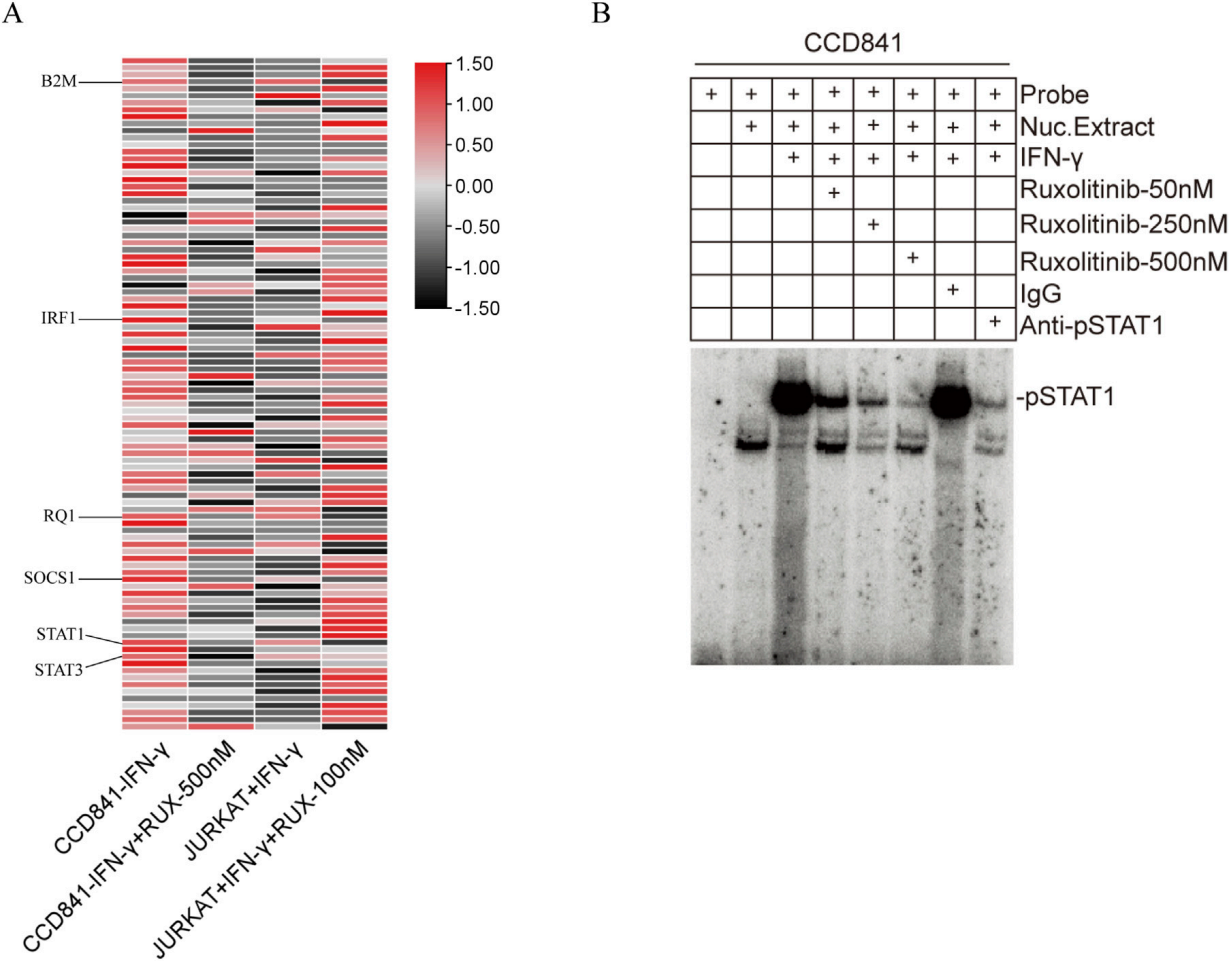
Effect of ruxolitinib on JAK/STAT signaling pathway-related genes and inhibition of interferon (IFN)-γ-Induced STAT1 activation by ruxolitinib. **(A)** Heatmap of transcription levels (fold-changes) of the JAK/STAT signaling-related genes in CCD841 and Jurkat cells after treatment with IFN-γ and ruxolitinib. **(B)** Nuclear extracts prepared from CCD841 cells with or without IFN-γ activation and analyzed for STAT1 activation using electrophoretic mobility shift assay (EMSA) with the STAT1-binding consensus sequence DNA probe. Ruxolitinib at 50, 250, and 500 nM was incubated with STAT1-DNA complexes. RUX: ruxolitinib.

To assess the effects of Ruxolitinib on the JAK/STAT signaling pathway, we used the RT^2^ Profiler™ PCR Array to detect the expression levels of 96 genes. Gene expression was analyzed using the ΔΔCT method and visualized as a heatmap (Figure 4A). Several genes showed significant upregulation (Fold Change >1.5) following IFN-γ stimulation in both CCD841 and Jurkat cells, which were subsequently downregulated after Ruxolitinib treatment. These genes included B2M, IRF1, RQ1, SOCS1, STAT1, and STAT3. The Fold Change values of these genes, compared to the untreated control group, are summarized in Table 2.

**TABLE 2:** Fold change values of significantly differentially expressed genes in the JAK/STAT signaling pathway after IFN-γ pre-treatment in CCD841 and Jurkat cells with or without Ruxolitinib intervention.

Gene Name	CCD841			JURKAT		
	Control	IFN-γ	IFN-γ+RUX-500nM	Control	IFN-γ	IFN-γ+RUX-100nM
B2M	1	2.66	1.21	1	2.78	0.96
IRF1	1	9.33	1.21	1	2.67	1.23
RQ1	1	2.61	1.21	1	2.36	0.89
SOCS1	1	5.84	1.19	1	2.67	0.96
STAT1	1	5.94	1.19	1	3.85	0.52
STAT3	1	1.92	0.74	1	1.61	1.51

IFN-γ, interferon-γ; RUX, ruxolitinib.

### 3.5 Ruxolitinib inhibits IFN-γ-induced STAT1 activation

IFN-γ is a well-known activator of the JAK/STAT signaling pathway. Based on the PCR array results from the previous step, we hypothesized that Ruxolitinib might inhibit the activation of its downstream target, STAT1. To test this hypothesis, we performed an Electrophoretic Mobility Shift Assay (EMSA) to examine the interaction between proteins and DNA using a STAT1 binding consensus sequence probe. The formation of a protein-DNA complex was detected as a distinct shift in the gel, indicating binding.

The results showed that, compared to the unstimulated group, IFN-γ treatment significantly increased the STAT1 content in the nuclear extracts. Moreover, the positive control, anti-STAT1 antibody, effectively inhibited most of the STAT1 activation, whereas normal IgG had no such effect. In the presence of Ruxolitinib, we observed that 50 nM of Ruxolitinib was able to inhibit a significant portion of STAT1 activation, and 500 nM nearly completely suppressed STAT1 activation. This effect was comparable to that of anti-STAT1 antibody. These findings indicate a dose-dependent inhibition of STAT1 activation, as evidenced by a reduction in the binding intensity in the EMSA assay (Figure 4B). Together, these results demonstrate that Ruxolitinib can effectively block IFN-γ-induced STAT1 activation in a dose-dependent manner.

### 3.6 Ruxolitinib inhibits STAT1 phosphorylation in immune cells and normal intestinal epithelial cells

**FIGURE 5:**
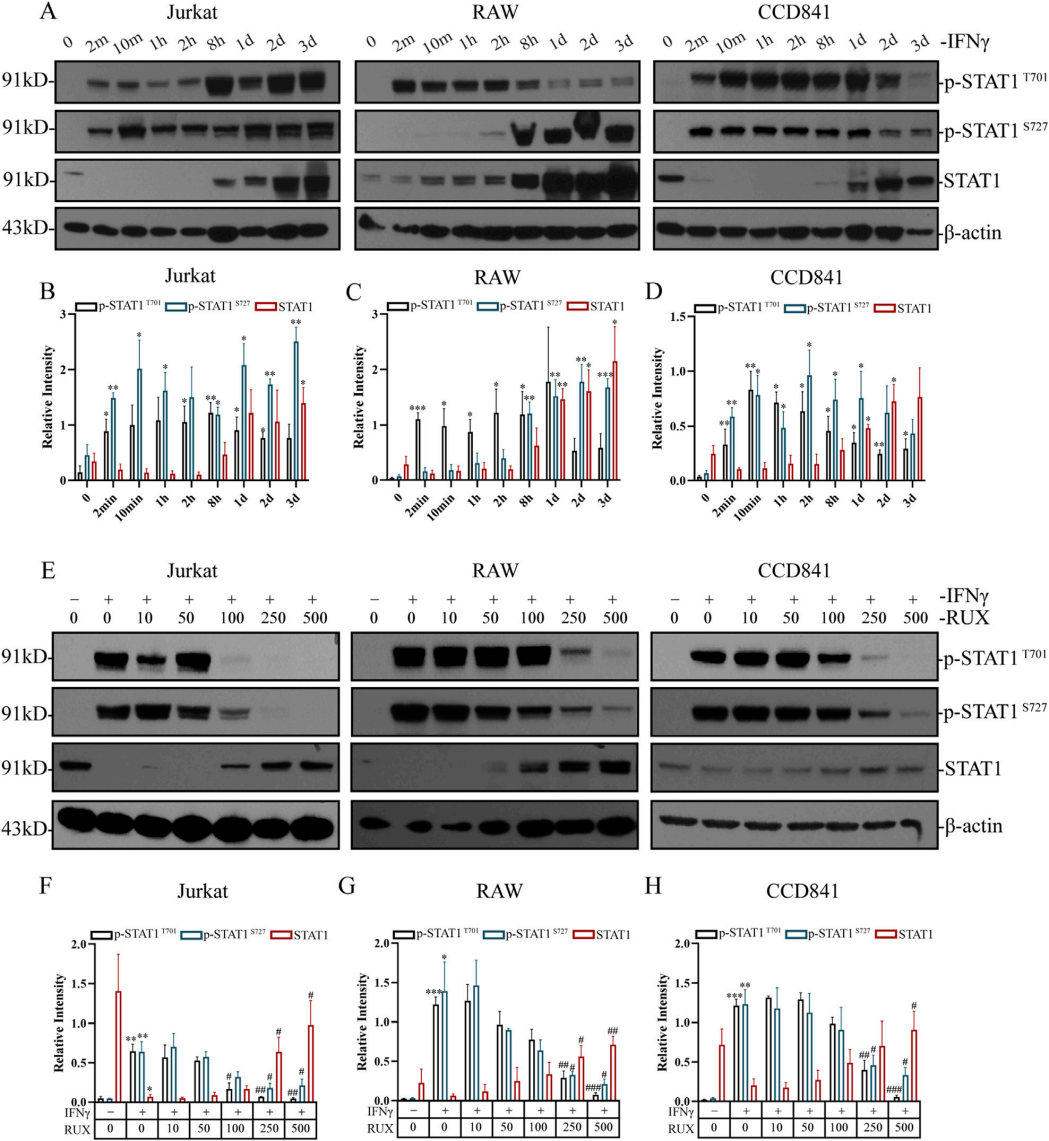
Inhibition of STAT1 phosphorylation by ruxolitinib in immune cells and normal intestinal epithelial cells. **(A)** Western blot analysis of p-STAT1Y701, p-STAT1S727, STAT1, and β-actin in Jurkat, RAW and CCD841 cells treated with 50 IU/mL IFN-γ for the indicated times. **(B–D)** Quantitative analysis of p-STAT1Y701, p-STAT1S727, STAT1 levels in **(B)** Jurkat, **(C)** RAW, and **(D)** CCD841 cells from **(A)**. **(E)** Western blot analysis of p-STAT1Y701, p-STAT1S727, STAT1, and β-actin in cells pretreated with IFN-γ (8 h) followed by Ruxolitinib (RUX, 24 h) at indicated concentrations (μM). **(F–H)** Quantitative analysis of p-STAT1Y701, p-STAT1S727, STAT1 levels in **(F)** Jurkat, **(G)** RAW, and **(H)** CCD841 cells from **(E)**. # *P* < 0.05, ## *P* < 0.01, ### *P* < 0.001 vs. 0 μM RUX (IFN-γ-treated). Data are presented as mean ± SEM. *P < 0.05, **P < 0.01, *P < 0.001 vs. the group with IFN-γ concentration at 0; #*P* < 0.05, ##*P* < 0.01, ###*P* < 0.001 vs. the IFN-γ-treated group with RUX concentration at 0. RUX: ruxolitinib.

Western blot analysis demonstrated that IFN-γ stimulation for 2 min significantly induced STAT1 phosphorylation at Tyr701 in human T cells (Jurkat), mouse macrophages (RAW), and human normal intestinal epithelial cells (CCD841), and at Ser727 in Jurkat and CCD841. In contrast, 8 h stimulation significantly induced STAT1 phosphorylation at Ser727 in RAW cells (Figure 5A). In RAW and CCD841 cells, the levels of p-STAT1 ^T701^ initially increased but decreased after reaching a plateau phase. Similarly, in CCD841 cells, the levels of p-STAT1 ^S727^ initially increased but decreased after plateauing. After IFN-γ treatment, STAT1 protein levels were upregulated in all three cell types and remained elevated. Grayscale analysis of the Western blot results revealed that both p-STAT1 ^T701^ and p-STAT1 ^S727^ levels significantly increased in 2 min following IFN-γ treatment, with more pronounced differences observed after 8 h in all three cell types. STAT1 levels, on the other hand, showed significant elevation after 1 or 2 days (Figures 5B–D).

To evaluate the effect of Ruxolitinib on STAT1 phosphorylation, cells were treated with varying concentrations of Ruxolitinib following 8 h of IFN-γ stimulation. As observed in previous experiments, IFN-γ alone markedly increased p-STAT1 levels at both Tyr701 and Ser727 in all three cell lines, while STAT1 expression decreased significantly only in Jurkat cells. Treatment with Ruxolitinib showed that 100 μM effectively inhibited STAT1 phosphorylation at Tyr701 and 250 μM effectively inhibited phosphorylation at Ser727 in Jurkat cells. In contrast, a concentration of 250 μM was necessary to achieve similar effects in RAW and CCD841 cells at both p-STAT1 ^T701^ and p-STAT1 ^S727^ (Figure 5E). Grayscale analysis of the Western blot data revealed that Ruxolitinib intervention significantly reduced IFN-γ-induced p-STAT1 ^T701^ levels at concentrations of 100 μM (in Jurkat) and 250 μM (in Raw and CCD841). Similarly, Ruxolitinib intervention (250 μM) significantly reduced IFN-γ-induced p-STAT1 ^S727^ levels in all three cell lines. Furthermore, Ruxolitinib significantly increased STAT1 protein levels at concentrations of 250 μM (in Jurkat and RAW) and 500 μM (in CCD841) (Figures 5F–H). These findings suggest that Ruxolitinib effectively inhibits STAT1 phosphorylation in both immune and intestinal epithelial cells in a dose-dependent manner.

## 4 Discussion

Ulcerative colitis (UC) is a chronic inflammatory bowel disease that significantly impacts patients’ quality of life. Currently, treatment primarily relies on immunosuppressants, corticosteroids, and biologics (Liang et al., 2024). However, many patients experience suboptimal responses to these treatments, highlighting the need for more effective therapeutic options. Myeloid-derived suppressor cells (MDSCs) have been shown to play a critical role in UC pathogenesis. Transcriptomic studies on intestinal and blood samples from UC patients have identified MDSCs as key immune cells that are closely regulated by the JAK/STAT signaling pathway and are abnormally recruited to areas of colon damage. Ruxolitinib, a JAK1 inhibitor, is approved for the treatment of myelofibrosis (Zhang et al., 2022) and acute graft-versus-host disease (Malard et al., 2023) and has been demonstrated to regulate the immune microenvironment by inhibiting myeloproliferation. However, its potential in the treatment of UC remains underexplored. This study investigated the therapeutic potential of the JAK1/2 inhibitor Ruxolitinib in a DSS-induced acute colitis mouse model, focusing on its effects on disease activity, inflammatory responses, and the JAK/STAT signaling pathway.

Our results demonstrate that Ruxolitinib significantly improved disease activity and alleviated intestinal features of UC in the mouse model. Treatment with Ruxolitinib significantly reduced weight loss and notably improved the DAI score during the later stages of treatment, as supported by Li C. et al. (2023), who also showed that ruxolitinib improved intestinal barrier function, decreased intestinal cell apoptosis, and improved mouse survival. These findings suggest that Ruxolitinib can potentially alleviate UC by reducing systemic inflammation and slowing disease progression. Additionally, Ruxolitinib treatment improved the shortened colon length induced by DSS, significantly reduced spleen enlargement, and mitigated intestinal mucosal damage, indicating that it can alleviate both systemic and localized inflammatory responses. These results further support the idea that Ruxolitinib effectively addresses the inflammatory processes in UC.

In pathological conditions, MDSCs proliferate significantly and are recruited to inflammation sites, where they suppress T cell activity, induce the Treg phenotype (Pang et al., 2020), inhibit NK cell cytotoxicity (Tong et al., 2022), and modulate macrophage polarization via TGF-β and IL-10 (Wang et al., 2023). These immune regulatory mechanisms contribute to suppressed adaptive immune responses and exacerbate inflammation. Previous studies have shown that modulating MDSC function can improve pathological features in experimental colitis models (Xun et al., 2023; Yun et al., 2022). In this study, flow cytometry results showed that Ruxolitinib treatment significantly reduced the proportion of MDSCs in the blood of colitis mice compared to the model group. This parallels its effects in bone marrow failure where it rebalances Treg/effector T-cell populations (Aggarwal et al., 2023), suggesting conserved immunomodulatory properties across disease contexts. Mechanistically, we propose that ruxolitinib attenuates MDSC expansion and activation through JAK/STAT pathway inhibition, thereby restoring immune competence and mitigating tissue injury.

The JAK/STAT signaling pathway plays a crucial role in inflammatory diseases and intestinal inflammation-associated tumorigenesis. IFN-γ, a cytokine in peripheral blood, can activate this pathway and induce MDSC generation (Cai et al., 2021; Li Y. et al., 2023), which in turn suppresses T cell activation. Based on this, we pre-treated immune and normal intestinal epithelial cells with IFN-γ to activate the JAK/STAT signaling pathway and observe the inhibitory effect of Ruxolitinib. PCR array analysis identified several key genes, including B2M, IRF1, RQ1, SOCS1, STAT1, and STAT3, which were significantly upregulated by IFN-γ and significantly downregulated after Ruxolitinib treatment. Notably, the downregulation of STAT1 expression was particularly pronounced. To further validate these findings, we employed the EMSA assay and demonstrated that Ruxolitinib effectively blocked IFN-γ-induced STAT1 activation *in vitro*. Inhibiting the phosphorylation of STAT1 has been shown to weaken the immunosuppressive functions of MDSCs (zhang et al., 2024), which supports our experimental findings that Ruxolitinib can modulate the transcription of genes associated with the JAK/STAT1 signaling pathway. Moreover, Western blot analysis further confirmed that Ruxolitinib inhibited IFN-γ-induced phosphorylation of STAT1 at both Tyr701 (T701) and Ser727 (S727) residues. Critically, these phosphorylation events serve distinct functional roles: phosphorylation at Tyr701 is essential for STAT1 dimerization, nuclear translocation, and DNA binding, while phosphorylation at Ser727 enhances its transcriptional activity (Levy and Darnell, 2002). Ruxolitinib’s effective suppression of both phosphorylation sites suggests comprehensive modulation of the STAT1 signaling pathway. This dual inhibition likely contributes to the observed downregulation of STAT1-dependent genes and attenuation of downstream inflammatory responses. Li C. et al. (2023) also showed that the effects of ruxolitinib in UC required the expression of STAT3. MDSCs accumulate in inflammatory microenvironments (Kim et al., 2025), and JAK/STAT signaling enhances the expansion and immunosuppressive function of MDSCs (Cao et al., 2023). Therefore, it is an innovative approach to studying the JAK/STAT signaling molecules and their modulation by ruxolitinib in UC models. This study elucidated the treatment potential of ruxolitinib using *in vivo* and *in vitro* models by exploring the blood MDSC population and JAK/STAT signaling. The results help us understand the mechanism of action of ruxolitinib in UC.

This study also had some limitations. First, UC in humans is a chronic inflammatory disease, while the DSS-induced UC model is acute. Chronic inflammatory responses differ from acute inflammatory responses. Therefore, a UC chronic inflammatory model needs to be designed and used to elucidate UC pathogenesis and the effect of treatment. Second, clinical use of JAK inhibitors carries established risks of opportunistic infections (e.g., herpes zoster reactivation) and hematological adverse events (Mansilla-polo and Morgado-Carrasco, 2024). Our 14-day intervention period did not assess these critical safety parameters, nor did it evaluate potential long-term hematopoietic toxicity.

## 5 Conclusion

In conclusion, our findings suggest that Ruxolitinib has potential therapeutic applications in UC by modulating the JAK/STAT1 signaling pathway, inhibiting MDSC activation, and alleviating inflammatory responses. These insights provide a better understanding of the mechanisms of Ruxolitinib in UC and highlight its potential as a novel therapeutic strategy for UC. Future studies should focus on the long-term effects and safety of Ruxolitinib in clinical settings, as well as its potential in combination with other immune modulators to enhance its therapeutic efficacy.

## Data Availability

The original contributions presented in the study are included in the article/supplementary material, further inquiries can be directed to the corresponding author.
